# Anti-SIRT1 autoantibody is elevated in ankylosing spondylitis: a potential disease biomarker

**DOI:** 10.1186/s12865-018-0280-x

**Published:** 2018-12-17

**Authors:** Qiongyi Hu, Yue Sun, Yuan Li, Hui Shi, Jialin Teng, Honglei Liu, Xiaobing Cheng, Junna Ye, Yutong Su, Yufeng Yin, Mengru Liu, Jiucun Wang, Chengde Yang

**Affiliations:** 1 Department of Rheumatology and Immunology, Ruijin Hospital, Shanghai Jiao Tong University School of Medicine, No. 197 Ruijin Second Road, Shanghai, 200025 China; 2 State Key Laboratory of Genetic Engineering and Ministry of Education (MOE) Key Laboratory of Contemporary Anthropology, Collaborative Innovation Center for Genetics and Development, School of Life Sciences, Fudan University, Shanghai, 200438 China

**Keywords:** AS, Anti-SIRT1 antibodies, Biomarker

## Abstract

**Background:**

Little is known about the presence of specific autoantibodies in ankylosing spondylitis (AS), an immune-mediated inflammatory disease. The object of this study was to explore potential autoantibody profiles in AS patients.

**Results:**

Levels of anti-SIRT1 autoantibodies were significantly higher in AS (*P <* 0.001) and psoriatic arthritis (PsA) (*P* < 0.01) patients but not rheumatoid arthritis (RA) patients compared with healthy controls. Additionally, titers of anti-NAD-dependent protein deacetylase sirtuin-1(SIRT1) antibodies were significantly higher in AS patients than in RA (*P* < 0.05) and PsA (*P* < 0.05) patients. Moreover, levels of anti-SIRT1 (*P* < 0.001) antibodies were significantly higher during the first year in patients with hip joint involvement. The anti-SIRT1 antibody positivity rate was 18.9% in AS patients.

**Conclusions:**

Our findings indicate that anti-SIRT1 autoantibodies may serve as a marker for diagnosing AS and predicting hip joint involvement at an early stage.

**Electronic supplementary material:**

The online version of this article (10.1186/s12865-018-0280-x) contains supplementary material, which is available to authorized users.

## Background

Ankylosing spondylitis (AS) is an immune-mediated, insidiously progressive form of seronegative spondyloarthritis (SpA) that is characterized by enthesitis. AS progressively leads to inflammation, bone erosion, new bone formation and ankylosis of sacroiliac, vertebral, and peripheral joints, with the ultimate radiographic appearance of a “bamboo spine” [1]. The pain and ankylosis that occur in this disease may cause considerable disability.

Although the etiology of AS is incompletely understood, familial and genetic factors are thought to be involved [2]. A surge of genome-wide association studies (GWASs) have been performed to identify associations between genetic variants and AS phenotypes, including the human leukocyte antigen allotype B27 (HLA-B27), the major risk factor for AS, as well as non-MHC genetics [2]. By delineating potential immunomodulatory pathways involving regulating innate and acquired immunity, such GWASs have provided considerable evidence for understanding the pathogenesis of AS [3]. The role of HLA-B27 in antigen presentation to T cells has been well established, and the association of *IL-7R, ZMIZ1, EOMES* and *RUNX3* variants with AS suggests the involvement of CD8^+^ T cell-mediated immunity [4]. Regardless, elucidating the roles of these factors in the immune system requires further study. Moreover, elevated numbers of Th17 cells and levels of IL-23 expression have been found in the peripheral blood of AS patients, indicating that CD4^+^ T cell-mediated inflammation contributes to the pathogenesis of AS. In addition to chronic inflammation, cartilage degeneration and new bone formation are key pathogenic features of AS. It has also been suggested that enhanced bone morphogenetic protein (BMP) and Wnt/β-catenin signaling contributes to ankylosis and chondrogenesis in AS [5].

Despite sharing a susceptibility locus HLA genotype with other autoimmune diseases such as systemic lupus erythematosus (SLE) and Sjögren syndrome (SS) [6, 7], it remains controversial whether AS is an autoimmune disease with specific autoantibodies. Nonetheless, specific immune complexes have recently been found to be involved in AS pathogenesis [8], and levels of antibodies against connective, skeletal, and muscular tissue-related antigens [9], PPM1A [10], CD74 [11, 12], leukocytes [13], neutrophils [14], and some collagen proteins [15] are high in AS patients. Furthermore, an increased prevalence of anti-glycan antibody has been noted in AS and psoriatic arthritis (PsA) patients, with rheumatoid arthritis (RA) patients showing even higher prevalence [16]. In another study, AS patients were found to have higher levels of anti-flagellin antibody than a control group, suggesting an immune response to bacterial antigens in AS patients [17]. A comprehensive review on novel diagnostic autoantibodies in AS has been published, and targets of autoantibodies in AS include microbes, inflammatory factors and structural antigens [18]. Although these studies reveal a certain spectrum of autoantibodies in AS, the AS-related antibodies reported to date were based on small-study populations, and further validation is lacking. More evidence is needed to ascertain the autoreactivity associated with AS.

AS is considered an inflammatory rheumatoid disease, yet it differs from other autoimmune diseases with specific autoantibodies such as SLE and SS, and definitive evidence for autoantibodies in AS is lacking. The aim of this study was to explore potential autoantibody profiles in AS patients using a protein microarray expressing 19,349 recombinant human proteins. Autoantibodies targeting NAD-dependent protein deacetylase sirtuin-1 (SIRT1) in the protein microarray were then validated by an ELISA-based method, and significant differences in anti-SIRT1 antibody levels in AS patients with different clinical variables were assessed.

## Results

### Global properties of observed antibodies in AS

**Fig. 1:**
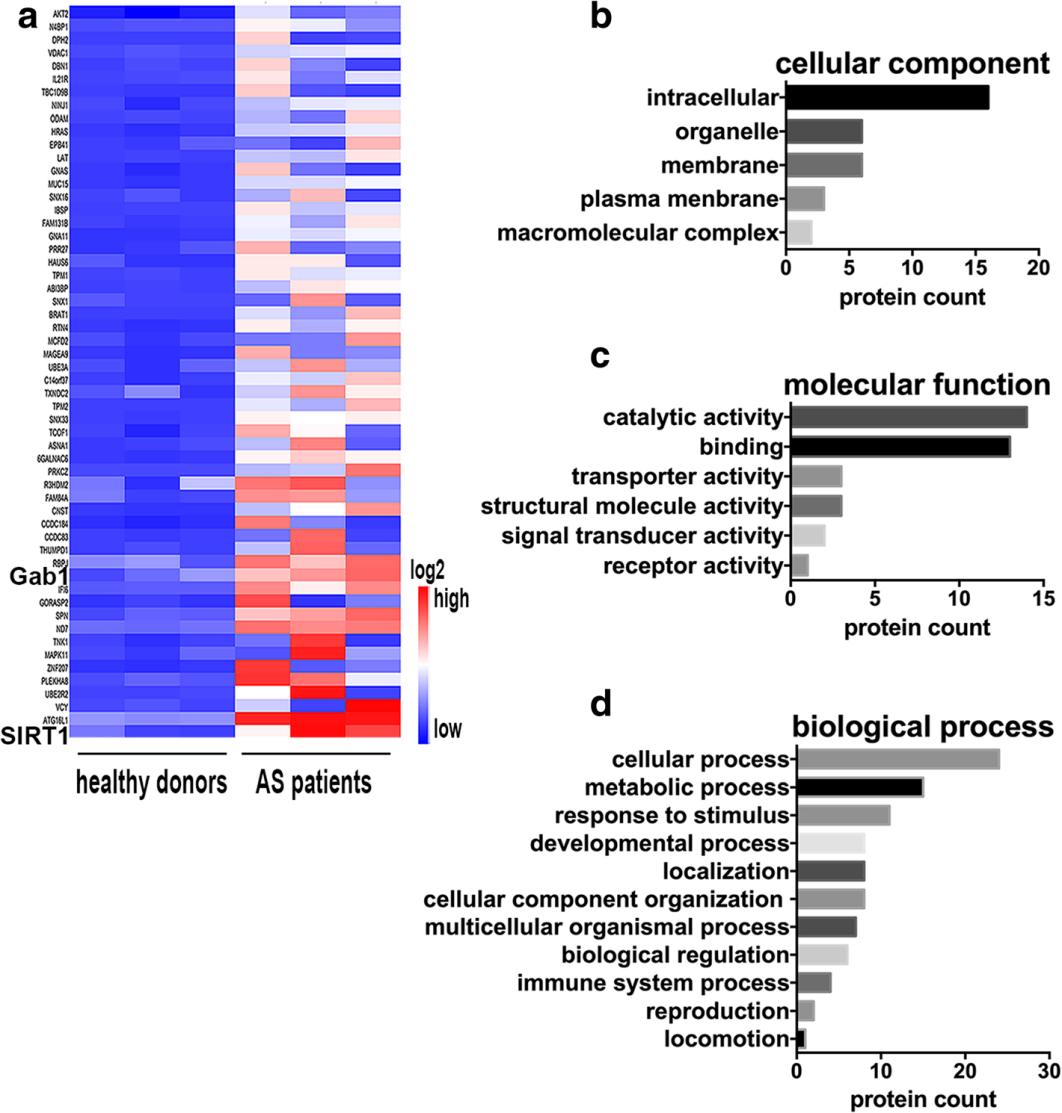
Proteomic analysis of sera from AS patients. **a**. Heat map representing the 56 protein candidates targeted by IgG antibodies in AS patient sera with 4-fold or greater expression (*P <* 0.05) compared with healthy donors. The classification and clustering of different categories of cellular component (**b**), molecular function (**c**) and biological process (**d**) of all identified proteins were analyzed using PANTHER databases

First, we explored global antibody profiles in serum from AS patients using a protein microarray displaying 19,349 immobilized recombinant human proteins. Analysis of the protein array data revealed 56 targets among the 2125 IgG antibodies expressed at levels 4-fold or greater in AS patients compared with healthy donors (Fig. 1a and Additional file 1: Table S1). Interestingly, functional analysis using the PANTHER pathway classification system indicated that the targets are mainly intracellular (Fig. 1b), similar to autoimmune diseases such as SLE. Additionally, the most significant enrichment in the antibody signature of AS patients was for terms of “catalytic activity” and “binding” (Fig. 1c). Further analysis of biological processes showed that most of these proteins are related to cellular, metabolic, stimulus response and developmental processes (Fig. 1d). Moreover, 13 proteins targeted by IgG antibodies were more than 10-fold higher in AS patients. The functions of 13 proteins were listed in Table 1. SIRT1 was the only antigen for which there was a significant highest level of IgG antibody in serum from AS patients compared to healthy controls. Interestingly, SIRT1 has been demonstrated to regulate bone metabolism; therefore, we concentrated on SIRT1 as a target.

**Table 1:** Targets of IgG antibodies with 10-fold or greater in AS patients

Name	Fold change	Functions
SIRT1	29.3	NAD-dependent protein deacetylase sirtuin-1
ATG16L1	26.4	Autophagy
VCY	23.0	Spermatogenesis
UBE2R2	20.5	Ubiquitin-conjugating enzyme
PLEKHA8	19.9	Cargo transport protein
ZNF207	16.8	Kinetochore- and microtubule-binding protein
MAPK11	16.3	MAP kinase signal transduction pathway
TNK1	14.9	Negatively regulating RAS-MAPK pathway
ND7	14.7	NADH dehydrogenase subunit 7
SPN	14.3	Regulating multiple T-cell functions
GORASP2	13.1	Golgi reassembly-stacking protein 2
IFI6	11.6	Regulating apoptosis, anti-viral activity
GAB1	11.1	GRB2-associated-binding protein 1
RBPJ	11.0	Playing a central role in Notch signaling
THUMPD1	10.6	tRNA acetylation
CCDC83	10.2	Coiled-coil domain-containing protein
CCDC184	10.1	Coiled-coil domain-containing protein
CNST	10.0	Required for targeting of connexins to the plasma membrane

### Distinct antibody expression levels in common rheumatic diseases

**Fig. 2:**
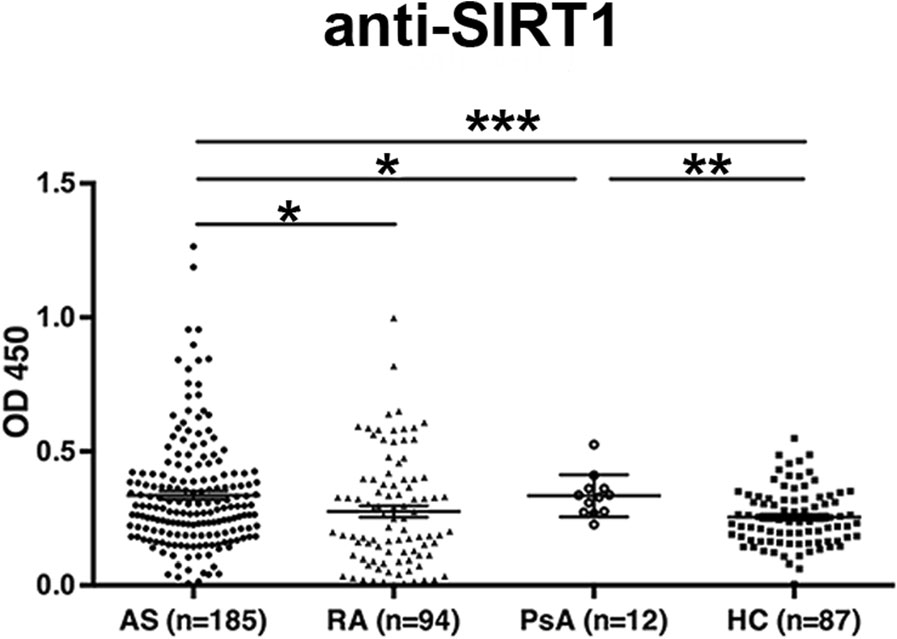
Serum anti-SIRT1 antibody levels in patients with AS, RA, and PsA as well as healthy controls were determined by ELISA. * *P* < 0.05, **P < 0.01, *** *P* < 0.001

To confirm the proteomic results, sera from 185 AS patients, 94 RA patients, 12 PsA patients, and 87 healthy controls were collected. The clinical characteristics of the subjects in each group are detailed in Table 2. As shown in Fig. 2, anti-SIRT1 antibody levels were significantly higher in AS patients than in healthy controls (*P <* 0.001). Among the disease controls, anti-SIRT1 antibody levels in AS patients were significantly different from those in RA (*P* < 0.05) and PsA (*P* < 0.05) patients (Fig. 2). Moreover, sera from 35 patients with AS (18.9%) and 18 with RA (19.1%) were positive for IgG antibodies against SIRT1, whereas serum from only 1 patient with PsA (1%) was positive for anti-SIRT1 antibodies.

**Table 2:** Characteristics of the study cohort

Variable	AS	RA	PsA	HC
Number	185	94	12	87
Age (years) (median)	15–70 (34.6)	14–54 (41.1)	31–62 (45.1)	20–50 (35.2)
Gender ratio (male: female)	139:46	12:82	10:2	66:21
Disease duration (years)	7.4 (0.04–31)	–	–	–
Positive for HLA-B27	94.3%	–	–	–
Hips joint involvement (n, %)	57 (30.8%)	0	0	–
ESR (mm/h) (median)	31.6 ± 42.3	31.2 ± 25.7	32.6 ± 25.9	–

### Anti-SIRT1 antibody levels were higher in female AS patients

**Fig. 3:**
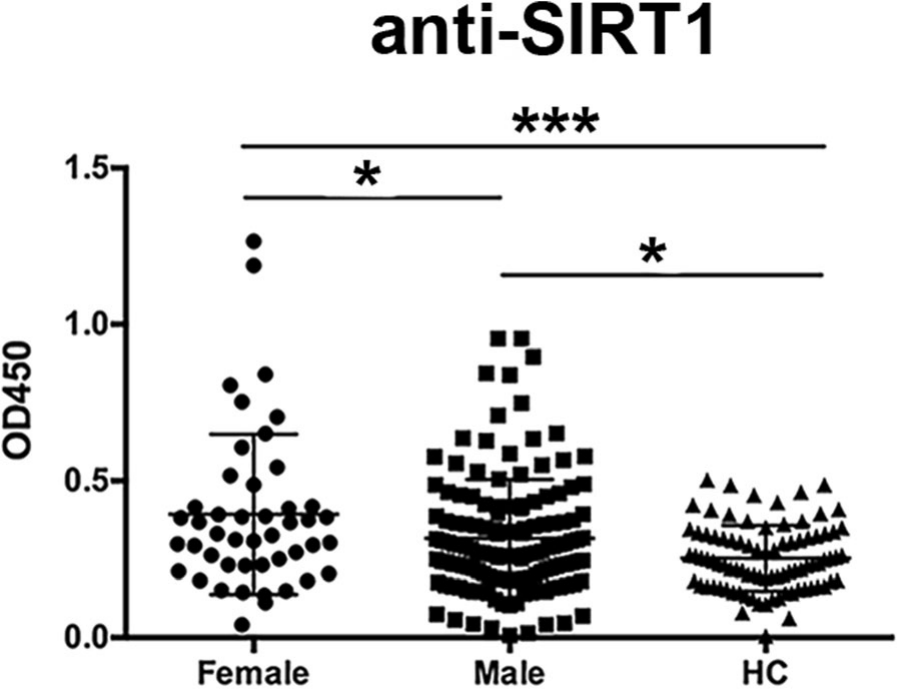
Higher levels of anti-SIRT1 antibodies in female AS patients than in male AS patients and healthy controls. *P < 0.05, *** *P* < 0.001

As sex hormones contribute to many autoimmune diseases including SLE, SS and AS [19], we compared levels of anti-SIRT1 antibodies between male and female AS patients. Remarkably, levels of IgG antibodies against SIRT1 were significantly higher in sera from female patients compared to male patients (Fig. 3, *P* < 0.05). However, no gender difference for anti-SIRT1 antibody levels was observed among RA and PsA patients (data not shown). These results indicate that sex hormones may modulate the production and secretion of antibodies in AS patients.

### Elevated levels of anti-SIRT1 autoantibodies in AS patients with hip joint involvement at the early stage

**Fig. 4:**
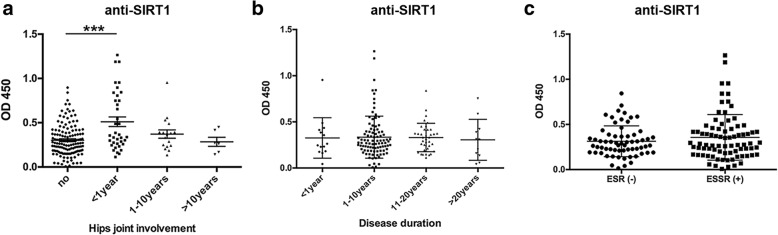
Serum levels of anti-SIRT1 antibodies in AS patients with hip joint involvement, various durations of disease and ESR levels. **a**. Serum anti-SIRT1 antibodies in AS patients without hip joint involvement and with involvement duration of < 1 year, 1–10 years and > 10 years from symptom onset. **b**. Serum anti-SIRT1 antibody levels in patients with AS duration of < 1 year, 1–10 years, 11–20 years and > 20 years from symptom onset. **c**. No significant differences in serum anti-SIRT1 antibody levels in ESR-positive and ESR-negative groups were found. *** *P* < 0.001

We next analyzed statistical differences in serum antibody levels in AS patients with different disease variables. As shown in Fig. 4a, levels of anti-SIRT1 antibodies (*P* < 0.001) were significantly higher in patients with hip joint involvement during the first year, though anti-SIRT1 antibody levels were similar among AS patients regardless of disease duration (Fig. 4b). Furthermore, anti-SIRT1 levels were not significantly different between ESR-positive and -negative patients and did not correlate with ESR levels (Fig. 4c).

## Discussion

In this study, we found serum levels of anti-SIRT1 antibodies to be significantly higher in sera from individuals with AS than from individuals with RA or PsA. In addition, levels of anti-SIRT1 antibodies were significantly higher in patients with hip joint involvement during the first year. The anti-SIRT1 antibody positivity rate among AS patients was 18.9%, suggesting that anti-SIRT1 antibody is a potential novel biomarker in AS.

Although the pathogenesis of AS is not fully understood, it is commonly recognized that genetic susceptibility, environmental factors (especially the microbiome) and the immune system may be crucial factors in the development of this disease [20]. Indeed, multiple genetic variations have been found in genes encoding proteins essential for antigen presentation and macrophage-related phagocytosis, such as *UBE2E3, ERAP1* and *FCGR2A* [1]. These data suggest that dysfunctional antigen processing and eradication occurs in AS. To date, a few studies have identified targets of autoantibodies in AS [9–15], though more definite evidence needs to be provided to verify the existence of autoreactivity in AS patients.

As imbalanced ossification is a hallmark in AS, we focused on proteins related to bone metabolism among 13 proteins targeted by IgG antibodies and showing more than 10-fold increase in AS patients. Interestingly, the anti-SIRT1 antibody level was highest in serum from AS patients. SIRT1, a widely distributed class III histone deacetylase, is involved in regulating T cell activation as well as tolerance and inflammation [21, 22]. Additionally, recent studies have indicated a potential role for SIRT1 in regulating bone metabolism [23], and reduced levels of SIRT1 in osteoblasts from patients with osteoarthritis, a disorder characterized by inappropriate osteogenesis of bone tissue, lead to increased expression of TGF-β and sclerostin, which regulates bone mineralization via Wnt signaling [24]. Furthermore, MSCs isolated from SIRT1-deficient mice exhibit impaired differentiation into osteoblasts and chondrocytes [25], the major cells involved in the pathogenesis of AS. Moreover, SIRT1 is a negative controller of NF-kB, and well-demonstrated dysregulation of the NF-kB pathway, which is crucial for inflammation and the survival of osteoclasts and osteoblasts, has been reported in AS [26]. In our study, we found elevated levels of anti-SIRT1 antibodies in patients with hip joint involvement during the first year, indicating that SIRT1 may regulate erosive bone destruction in AS. We will explore the pathogenic role of SIRT1 in AS in the future.

PANTHER analysis revealed intracellular proteins to be the main targets of autoantibodies in AS. SIRT1 is mainly a cytoplasmic protein, and the autoreactivity against SIRT1 found in AS serum might reflect failed elimination of these antigens. More research will need to explain the role of these antigens in AS, and we regret the unavailability of spinal samples from AS patients to determine local expression of SIRT1 at affected joints.

Interestingly, our results showed that anti-SIRT1 levels were significantly higher in female patients compared to male patients. For some autoimmune diseases, sex hormones are among the most-studied contributory factors [27]. Unlike other autoimmune diseases with higher prevalence in females, such as SLE, SS and RA, AS is more prevalent in males than in females, and male patients also have more severe disease than do female patients [28]. It has been reported that testosterone might interact with SIRT1 to protect endothelial cells [29], though it remains to be determined how testosterone regulates SIRT1 and whether it might affect autoantibody production in AS.

Despite the presentation of SIRT1 as a self-antigen in AS, there were no significant differences in antibody levels when comparing diverse groups with low and high ESR or those with different disease durations. This finding indicates that the autoantibodies determined in the current study are irrelevant to inflammation in AS. Considering the functions of SIRT1 in bone formation as well as the positive correlation regarding autoreactivity, our study provides a basis for further research of the pathogenic mechanism of the immune system in AS.

AS has been recognized as a seronegative disease due to the lack of specific autoantibodies, which are the immunologic hallmarks of many autoimmune diseases such as SLE, SS and RA [30]. The present study reveals unique characteristics of AS compared with autoimmune diseases such as RA.

## Conclusions

In summary, we found sera from AS patients to be extraordinarily distinct in terms of antibody reactivity toward antigens. In addition to the inflammatory response, autoantibodies produced by dysregulation of the immune system might constitute another feature of AS. Their core signature showed enrichment of a diverse array of proteins involved in bone metabolism, which may facilitate progression of AS, and included several novel antigens the function of which is not yet understood. These findings provide a framework for better definition of the role of the immune response and autoantibodies in the pathogenesis of AS. Moreover, we report the existence of anti-SIRT1 antibodies in sera from AS patients and the potential of anti-SIRT1 antibodies to serve as a disease biomarker for AS.

## Patients and methods

### Subjects

The first cohort of patients consisted of 10 treatment-naïve AS patients who fulfilled the modified 1984 New York criteria for AS [31]; 12 sex- and age-matched healthy donors were used as controls. Sera were collected and used for protein array analysis. The clinical characteristics of each group are summarized in Additional file 2: Table S2.

The second cohort of patients and controls consisted of 185 consecutive patients with AS, 94 patients with RA, 12 patients with PsA according to standard diagnostic criteria [32, 33]; 87 sex- and age-matched healthy donors were used as controls. Among the 185 AS patients and 87 healthy controls, sera from 10 AS patients and 12 healthy controls were used for protein array analysis. Sera from the second cohort were used for ELISA analysis. All of the serum samples were collected at Ruijin Hospital between 2015 and 2016 and stored at − 80 °C until use. The study was performed in accordance with the Declaration of Helsinki and the principles of Good Clinical Practice. Biological samples were obtained under a protocol approved by the Institutional Research Ethics Committee of Ruijin Hospital (ID: 2016–62), Shanghai, China. All participants provided informed consent to participate in the study. Demographic and laboratory data were obtained by the Department of Immunology and Rheumatology.

### Serum antibody profiling using a human protein microarray

The human proteome microarray (BC Biotechnology, USA) used in this study is composed of approximately 19,394 unique full-length recombinant proteins printed in duplicate. As a screening procedure, we employed protein array technology to detect new autoantibodies in AS using the first cohort of patients and healthy controls. Treatment-naïve patients with AS (*n* = 10) were divided into 3 groups, and sera of every group were mixed for detection. Sex- and age-matched sera from healthy donors (*n* = 12) divided into 3 groups were used as controls.

### Human protein microarray data analysis

The protein microarray data analysis was conducted according to the procedure described by Hu et al. [34]. Median foreground and background intensities were obtained for each spot in the protein microarrays using Gene Pix Pro 6.0 Software. To execute background correction and normalize intra-array signal intensity, the raw intensity of each spot was defined as the ratio of the foreground to the background median intensity. To remove the negative effects of non-specific binding and spatial heterogeneity across the protein microarray, “normexp” [35], a background processing approach, was implemented in the R 2.15.1 package for background correction. The *P* value was calculated for each protein using Fisher’s test, and fold change was defined as the mean signal intensity ratio of AS patients to healthy donors.

### Identifying proteins differentially recognized by serum autoantibodies

For each protein, spot signals were averaged across the groups. A statistical distribution of the signal intensities among the controls was calculated, from which the signal intensity mean and standard deviation were calculated. According to a previous autoantibody study by Meyer et al. [36], we identified the existence of 2125 proteins targeted by IgG antibodies by defining a cut-off of ≥2 SD from the mean signal intensity of healthy donors and imposing a minimum prevalence of 2/3 for the AS cases and a maximum prevalence of 1/3 for the healthy donors. After filtering the 2215 protein hits with *P* < 0.05 and fold changes of more than 4, 56 targets were identified as autoantigen candidates. Thus, for every target protein screened, the response of the control population formed a benchmark.

### Detection of serum autoantibodies

Serum levels of anti-SIRT1 were measured by ELISA. In brief, 96-well plates were coated with 1 μg/mL SIRT1 (R&D, Canada, USA) at 4 °C overnight. Nonspecific binding was blocked by incubating with PBS containing 5% BSA at 37 °C for 2 h. The wells were then incubated with human serum (1:100) at 37 °C for 2 h and washed five times with PBS plus 0.05% Tween-20. Secondary horseradish peroxidase (HRP)-conjugated goat anti-human IgG monoclonal antibodies (Jackson ImmunoResearch Laboratories, Inc., West Grove, PA, USA) diluted to 1:100,000 were added to each well. After five washes with PBS plus 0.05% Tween 20, 100 μL of tetramethybenzidine substrate solution (Sigma-Aldrich) was added, and the samples were incubated at room temperature. The reaction was terminated by the addition of 50 μL of 2 N H_2_SO_4_/well, and optical density (OD) was measured at 450 nm. A cut-off of ≥2 SDs of arbitrary units (AU) from the mean serum level of autoantibodies was used to qualitatively differentiate between positive and negative results.

### Statistical analyses

All data were analyzed statistically using SPSS version 20.0 (SPSS Inc., Chicago, IL, USA). Quantitative data are expressed as the mean ± SD. Differences between two groups were calculated using the Mann-Whitney U test or unpaired Student’s *t*-test, and differences between three or more groups were analyzed by one-way ANOVA and the Kruskal-Wallis test. Significant differences in antibody levels in AS patients with different clinical variables were evaluated using the Mann-Whitney U test or Kruskal-Wallis test. *P* < 0.05 was considered significantly different.

## Additional files

Additional file 1: **Table S1.** Targets of IgG antibodies in AS patients. (DOCX 16 kb)

Additional file 2: **Table S2.** Characteristics of AS patients and healthy donors in the protein microarray. (DOCX 14 kb)

## Abbreviations

AS: Ankylosing spondylitis
BMP: Bone morphogenetic protein
GWASs: Genome-wide association
HLA-B27: Human leukocyte antigen allotype B27
PsA: Psoriasis arthritis
RA: Rheumatoid arthritis
SIRT1: NAD-dependent protein deacetylase sirtuin-1
SLE: Systemic lupus erythematosus
SpA: Seronegative spondyloarthritis
SS: Sjögren syndrome
