# Supplementary material for: Anti-SIRT1 autoantibody is elevated in ankylosing spondylitis: a potential disease biomarker
Source: BMC Immunol. 2018 Dec 17;19:38. doi: 10.1186/s12865-018-0280-x (PMC6298004; doi:10.1186/s12865-018-0280-x)
Supplement: Supplementary file 1 — Table S1. Targets of IgG antibodies in AS patients. (DOCX 16 kb) [file 12865_2018_280_MOESM1_ESM.docx]

**Table** **S1 Targets of IgG antibodies in AS patients**

| **Name** | **Nucleotide ID** | **Fold change** |
| --- | --- | --- |
| SIRT1 | NM_012238.4 | 29.3 |
| ATG16L1 | NM_001363742.1 | 26.4 |
| VCY | NM_004679.2 | 23.0 |
| UBE2R2 | NM_017811.3 | 20.5 |
| PLEKHA8 | NM_032639.2 | 19.9 |
| ZNF207 | NM_001032293.1 | 16.8 |
| MAPK11 | NM_002751.5 | 16.3 |
| TNK1 | NM_001251902.1 | 14.9 |
| ND7 | BC041037.1 | 14.7 |
| SPN | NM_003123.3 | 14.3 |
| GORASP2 | NM_015530.3 | 13.1 |
| IFI6 | NM_002038.3 | 11.6 |
| GAB1 | NM_002039.2 | 11.1 |
| RBPJ | NM_015874.3 | 11.0 |
| THUMPD1 | NM_017736.3 | 10.6 |
| CCDC83 | NM_173556.4 | 10.2 |
| CCDC184 | NM_001013635.3 | 10.1 |
| CNST | NM_152609.2 | 10.0 |
| FAM84A | NM_145175.3 | 9.7 |
| R3HDM2 | BC041857.1 | 9.4 |
| PRKCZ | NM_002744.4 | 9.2 |
| ST6GALNAC6 | BC036102.1 | 8.6 |
| ASNA1 | BC002651.2 | 8.0 |
| TCOF1 | NM_001008657.1 | 7.8 |
| SNX33 | NM_153271.1 | 7.1 |
| TPM2 | NM_003289.3 | 7.0 |
| TXNDC2 | BC050132 | 7.0 |
| C14orf37 | NM_001001872.2 | 7.0 |
| UBE3A | BC002582.2 | 7.0 |
| MAGEA9 | BC002351 | 6.7 |
| MCFD2 | BC037845.1 | 6.4 |
| RTN4 | NM_207521.1 | 6.4 |
| BRAT1 | NM_152743.2 | 6.3 |
| SNX1 | NM_003099.3 | 6.2 |
| ABI3BP | NM_015429.2 | 6.1 |
| TPM1 | NM_000366.5 | 5.6 |
| HAUS6 | BC010632.1 | 5.5 |
| PRR27 | BX640997 | 5.5 |
| GNA11 | NM_002067.2 | 5.5 |
| FAM131B | BC050543.1 | 5.4 |
| IBSP | NM_004967.3 | 5.3 |
| SNX16 | NM_022133.2 | 5.1 |
| MUC15 | NM_145650.2 | 5.1 |
| GNAS | NM_016592.1 | 5.0 |
| LAT | NM_001014989.1 | 4.9 |
| EPB41 | BC039079.1 | 4.8 |
| HRAS | BC095471.1 | 4.7 |
| ODAM | BC017796.1 | 4.7 |
| NINJ1 | NM_004148.2 | 4.6 |
| TBC1D9B | BC008919.2 | 4.5 |
| IL21R | NM_021798.2 | 4.4 |
| DBN1 | NM_080881.1 | 4.2 |
| VDAC1 | NM_003374.1 | 4.1 |
| DPH2 | NM_001384.4 | 4.1 |
| N4BP1 | NM_153029 | 4.1 |
| AKT2 | NM_001626.4 | 4.0 |
