# Supplementary material for: Anti-SIRT1 autoantibody is elevated in ankylosing spondylitis: a potential disease biomarker
Source: BMC Immunol. 2018 Dec 17;19:38. doi: 10.1186/s12865-018-0280-x (PMC6298004; doi:10.1186/s12865-018-0280-x)
Supplement: Supplementary file 2 — Table S2. Characteristics of AS patients and healthy donors in the protein microarray. (DOCX 14 kb) [file 12865_2018_280_MOESM2_ESM.docx]

**Table S2** **Characteristics of AS patients and healthy donors in protein microarray**

| Variable | AS-A | AS-B | AS-C | All  AS patients | All  healthy donors |
| --- | --- | --- | --- | --- | --- |
| Number | 3 | 3 | 4 | 10 | 12 |
| Gender ratio (M: F) | 0:3 | 3:0 | 4:0 | 7:3 | 6:6 |
| Age (years) | 44 (27-43) | 28 (28-36) | 32 (21-44) | 35.3 (21-44) | - |
| Disease duration (years) | 2.75 ± 1.4 | 4.5 ± 2.1 | 6 ± 4.2 | 6.13 ± 4.25 | - |
| Positive for HLA-B27 | 100% | 100% | 100% | 100% | - |
| Hip involvement (n) | 0 | 0 | 2 |  | - |
| ESR (mm/h) (median) | 10.3 ± 6.5 | 43.3 ± 27.6 | 22.3 ± 36.0 | 25 ± 28.4 | - |
